# Epidemic Scarlatina

**Published:** 1848-05

**Authors:** 


### EPIDEMIC SCARLATINA.

A correspondent at Lawrenceburgh, Tennessee, mentions the prevalence in that neighborhood of a severe form of scarlatina in which, in many instances, the patients died in 22 and 24 hours. The disease had abated at the date of his letter, April 18th. In the fatal cases the brain was the organ which seemed especially to suffer.

